# All Your Base: a fast and accurate probabilistic approach to base calling

**DOI:** 10.1186/gb-2012-13-2-r13

**Published:** 2012-02-29

**Authors:** Tim Massingham, Nick Goldman

**Affiliations:** 1 European Bioinformatics Institute, Wellcome Trust Genome Campus, Hinxton, Cambridgeshire, CB10 1RQ, UK

## Abstract

The accuracy of base calls produced by Illumina sequencers is adversely affected by several processes, with laser cross-talk and cluster phasing being prominent. We introduce an explicit statistical model of the sequencing process that generalizes current models of phasing and cross-talk and forms the basis of a base calling method which improves on the best existing base callers, especially when comparing the number of error-free reads. The novel algorithms implemented in All Your Base (AYB) are comparable in speed to other competitive base-calling methods, do not require training data and are designed to be robust to gross errors, producing sensible results where other techniques struggle. AYB is available at http://www.ebi.ac.uk/goldman-srv/AYB/.

## Background

There can be little doubt that the vastly increased throughput of Next-Generation Sequencing (NGS) machines has revolutionised DNA sequencing, but the reads produced are both shorter and less accurate than those from capillary sequencing and discoveries from NGS are often verified using traditional sequencing [1]. The challenges to overcome to improve the accuracy and read length of NGS platforms are different from those that were faced by capillary sequencing [2] and require different strategies to tackle them. In particular, the phasing process -- individual molecules of DNA becoming out-of-step with others in the same cluster -- is complex and ultimately limits the length of reads which can be obtained from cluster-based sequencing-by-synthesis methods [2]. Here we develop an explicit statistical model of the sequencing process, including phasing and other signal-degrading processes. By implementing a base calling algorithm based on this model, our AYB software is able to produce more accurate reads.

Our statistical model is quite generic and so applicable to all sequencing-by-synthesis and similar platforms (sequencing-by-ligation, pyrosequencing; see Metzker [3] for a comparison) that rely on large numbers of clusters consisting of many homogeneous DNA molecules. We concentrate on the Illumina Genome Analyser (GA-II), both to provide a concrete foundation to aid exposition of the methods and because of local availability of data for testing and comparison. The mechanics of sequencing-by-synthesis on the GA-II platform have been described elsewhere in detail [3]. Here we present an overview of an idealised sequencing process to establish context and terminology for the rest of this paper and then a critique to show how errors arise.

Fragmented single-stranded DNA is washed through the lanes of a slide, where it attaches and is amplified to form a sequence-homogeneous cluster of molecules. Sequencing progresses in steps, referred to as cycles, with each cycle conceptually sequencing one position of DNA. For each cycle, a mixture of Fluorophore-Labeled Nucleotides (FLNs) is washed through the lanes of the slide and attach to the molecules in each cluster; attachment of more than one nucleotide in a given cycle is prevented by the presence of a reversible terminator element on each FLN. A different fluorophore is associated with each of the four nucleotides (A, C, G, T) and so the nucleotide sequence of the DNA fragments can be uniquely identified from the fluorescence. After the attachment process has run to completion, the intensity of fluorescence from each cluster is recorded in four channels, each channel being a combination of illumination with a specific laser and imaging through a specific filter. Clusters are artificially grouped into tiles, regions of the lane consistent over cycles, whose size is constrained by the capacity of the imaging equipment. The terminator elements and fluorophores are then cleaved from the FLNs, setting up each cluster so the FLNs in the next cycle will attach to the next position of sequence.

After processing the images to pick out individual clusters, the output of the sequencing machine is many *channel × cycle *matrices of intensities, one matrix for each cluster. In principle the bases could be called straight from these intensities but there are several complicating factors [4] that must be dealt with, cross-talk, phasing and dimming being of particular importance.

Cross-talk is the recording of light from a single fluorophore in multiple channels. This occurs because, although they are chosen to be distinguishable, the fluorophores' emission spectra overlap. There is not a one-to-one correspondence between channels and FLNs and the relationship between the emission of each fluorophore and the intensity observed in each channel needs to be ascertained and corrected for. Phasing refers to the deterioration in relationship between sequencing cycle and sequence position as the cluster loses coherence: on a given cycle, FLNs may be attaching to different positions on different molecules within the cluster. There are many possible explanations for phasing: for example, a FLN might have a defective reversible terminator element leading to the attachment of two FLNs to a molecule on a single cycle, allowing the molecule to get ahead in the sequencing process ('pre-phased'), or the cleaving of the reversible terminator might fail for a cycle so the molecule lags behind when the element is finally removed ('post-phased'). A further possible cause of post-phased molecules is the chemistry not running to completion, resulting in either no FLN being attached that cycle or cleaving failure as previously mentioned. Finally, molecules within a cluster gradually stop contributing to the total signal, possible causes being laser damage to the individual molecules or problems reversing the terminator element, and this leads to a decrease (dimming) in the overall emission observed from each cluster in later cycles of sequencing.

The cross-talk is a consequence of the physics of fluorophore excitation and methods for estimating it have already been developed for dye-terminated capillary electrophoresis sequencing platforms [5]. Phasing and dimming are more specific to NGS methods, the Illumina platform in particular, and have been approached in a variety of ways. The Illumina base calling software (Bustard) assumes a constant rate of post-phasing and pre-phasing for all cycles [6], as do the Alta-Cyclic caller [7] and Rolexa [8], whereas BayesCall [6] allows the phasing at each position of the sequence to depend on several of the neighbouring bases and Ibis [9] assumes that all information about the phasing at a given cycle is contained in intensities of the cycles either side. The Seraphim base-caller [10] assumes that cross-talk and phasing have already been corrected for, empirically estimating the sequencing noise and calling bases using a log-linear model over cycles to model base-specific signal degradation. In contrast our method uses a completely empirical model, generalising both cross-talk and phasing, that allows all aspects of the sequencing process to be determined by the data on a run-by-run, indeed tile-by-tile basis. This model is unique to AYB and incorporates effects such as cycle-wise variation in cross-talk and allows context-specific phasing rates that may account for some of the reported sequence specific errors in GC rich reads [11,12].

## Results and Discussion

In a recent comparison [4], Ledergerber and Dessimoz compared several different base callers and Ibis [9] was clearly the most accurate and considerably quicker than any comparable base caller. While Naïve Bayescall [13] is almost as accurate as Ibis and boasts a much improved base calling time over the original Bayescall algorithm [6] (37 min. compared to the 2266 min. for 300K reads, respectively, for Ledergerber and Dessimoz's example), it is still *c*. 24 × slower than Ibis and requires an already trained model to be available (Ledergerber and Dessimoz report 1842 min. for training). Despite its considerable improvement over the original algorithm, the computational requirements of Naïve Bayescall restrict its use and, since it is also strictly dominated by Ibis in both accuracy and speed, we do not include it in the comparisons below. To quantify the performance of AYB, we therefore use Bustard and Ibis as comparators to represent standard practice (Bustard; various versions depending on age of data) and the current state-of-the-art (Ibis; version 1.1.5).

Bustard is the standard base calling software for the Illumina platform and a detailed description of the algorithm is given in [6]. Ibis uses a machine learning approach to base calling, training a Support Vector Machine (SVM) at each cycle on the intensities of the current, previous and next cycles. The SVM is trained on true calls for some subset of the data, and these are estimated by mapping Bustard calls back to the reference; the trained SVMs are specific to given read lengths and cluster densities and may also incorporate artifacts that are specific to given run. If sequencing *de novo *then correct calls may not be available but an SVM from a similar run could be used, with potential reduced performance, or a small amount of a known genome could have been 'spiked-in' and the SVM trained using these reads. Ibis was trained by mapping the Bustard calls for a training set to the reference genome using the default aligner (a modified version of SOAP [14]), the training set comprising every tenth tile starting from the fifth for our full lane sets of data (*B. pert*., BGI and Illumina) and all reads for the reduced sets (*ϕ*X174 L2, *ϕ*X174 L4, *ϕ*X174 L6, Ibis Test and HiSeq; see below for full details of test data sets).

Here we compare the three base callers using six sets of data of varying read lengths, cluster densities and vintage typical of everyday use and of our extensive testing. The data sets are summarised in table 1 and may be freely obtained from the AYB website [15]. The callers were compared by both the percentage of reads that map to the reference genome and the percentage of reads that map back with no mismatches ('perfect' calls). The BWA short read aligner [16] was used to map reads back to the appropriate reference genome (edit distance of five) for all comparisons in this paper, having been chosen for its speed and its ability to deal with insertions and deletions.

**Table 1:** Summary of data sets analysed and reference genomes used for mapping.

Name	Reference genome	Num. reads	Read length	Paired-end	Date sequenced
*ϕ*X174 L2	*ϕ*X174	677538	76	no	Aug. 2008
*ϕ*X174 L4	*ϕ*X174	1299052	76	no	Aug. 2008
*ϕ*X174 L6	*ϕ*X174	900291	76	no	Aug. 2008
Ibis Test	*ϕ*X174	200000	51	no	Apr. 2009
*B. pert*.†	*B. pertussis *Tohama I	4250058	76	yes	Dec. 2009
BGI†	*H. sapiens *GRCh37	9611783	45	yes	Jul. 2008
Illumina†	*H. sapiens *GRCh37	13974025	51	yes	Jun. 2008
HiSeq	*H. sapiens *GRCh37 + *ϕ*X174	7813098	101	yes	Oct. 2010

The individual ends from paired-end sets are referred to with a suffix indicating the end, for example BGI/1, BGI/2. †These sets contain a whole lane; other sets have either been decimated (Ibis Test & HiSeq) or contain only a few tiles (*ϕ*X174 L2, *ϕ*X174 L4 and *ϕ*X174 L6). 'Date sequenced' is approximate, representing our best effort to quantify the vintage of the data. All intensity data are freely available from the AYB website [15] in the Illumina CIF format.

The following subsections describe features of each set of data and compare the performance of the base callers in more detail but a general summary is provided in table 2. Table 3 shows the time taken for both the model training and base calling steps where appropriate. Times are not given for Bustard since these calls are produced as part of the sequencing process and so are essentially free.

**Table 2:** Performance comparison of Bustard, Ibis and AYB on several sets of reads of varying read length and chemistry versions.

		Reads mapped, %					Reads perfect, %			
	Bustard	Ibis	Δ%	AYB	Δ%	Bustard	Ibis	Δ%	AYB	Δ%
*ϕ*X174 L2	76.62	78.33	+2.23	78.25	+2.13	55.88	58.94	+5.48	62.29	+11.48
*ϕ*X174 L4	63.02	66.11	+4.90	65.09	+3.29	40.09	43.08	+7.46	44.74	+11.60
*ϕ*X174 L6	72.09	74.07	+2.75	74.08	+2.77	51.19	53.34	+4.20	56.00	+9.40
Ibis Test	84.77	88.45	+4.34	88.19	+4.03	44.34	66.14	+49.16	69.32	+56.34
*B. pert*./1	28.76	39.16	+35.94	45.80	+58.98	2.53	3.14	+23.70	4.13	+62.86
... trimmed	77.35	81.06	+4.80	81.14	+4.90	39.52	47.64	+20.55	55.24	+39.79
*B. pert*./2	34.33	47.41	+38.75	53.50	+55.57	6.22	17.69	+183.98	26.67	+327.97
... trimmed	66.54	70.22	+5.53	72.07	+8.31	30.13	40.72	+35.18	48.25	+60.15
BGI/1	87.41	89.01	+1.82	88.85	+1.64	59.62	68.39	+14.70	69.29	+16.22
BGI/2	84.58	86.29	+2.03	86.52	+2.29	55.95	61.90	+10.64	63.30	+13.14
Illumina/1	97.58	97.80	+0.22	97.85	+0.28	72.55	75.80	+4.49	76.70	+5.73
Illumina/2	96.29	96.73	+0.46	96.82	+0.55	70.61	73.88	+4.63	74.66	+5.74
HiSeq/1	84.97	85.24	+0.32	85.97	+1.18	60.29	62.55	+3.75	64.50	+6.98
HiSeq/2	79.78	†		81.34	+1.76	49.79	†		55.58	+11.63

Performance is compared in terms of the percentage of reads mapped back to the reference with five edits or fewer, and the percentage of reads which perfectly match the reference; the 'Δ%' figures for Ibis and AYB, where given, are the percentage improvements over Bustard. See text for further details. †Ibis failed to process the second end of the HiSeq data.

**Table 3:** Training and base calling time for Ibis and AYB.

		Ibis		AYB	
	Training	Base calling	Total	Base calling	Δ%
*ϕ*X174 L2	68	2	70	12	-83
*ϕ*X174 L4	121	4	125	21	-83
*ϕ*X174 L6	114	3	117	15	-87
Ibis Test	6	4	10	1	-90
*B. pert*./1	21	13	34	119	+250
*B. pert*./2	33	21	54	163	+202
BGI/1	126	17	143	96	-32
BGI/2	117	29	146	96	-34
Illumina/1	136	27	163	196	+20
Illumina/2	136	41	177	197	+11
HiSeq/1	94	34	128	276	+116
HiSeq/2	†			274	

AYB does not require training, so only base calling time is reported. The Δ% figure for AYB is the percentage difference compared to the Ibis total time. All times are the real time, in minutes, running on four cores of an Intel Xeon L5420 running at 2.5 GHz. †Ibis failed to train on the second end of the HiSeq data so no time is reported.

### ϕX174, 76 cycle and 51 cycle

Two test sets from the bacteriophage *ϕ*X174 were used: nine tiles each from three lanes of 76 cycle data produced by the Sanger Institute and differing in cluster density (named *ϕ*X174 L2, *ϕ*X174 L4, *ϕ*X174 L6), and a decimated run containing 200K clusters of 51 cycle data that is distributed as a test set with Ibis [17] (Ibis Test). Reads from the 27 tiles from the Sanger Institute were aligned against a SNP-corrected genome (two SNPs corrected), such a correction being possible because of the high coverage produced by these tiles. Reads called from the Ibis Test set were mapped to the genome distributed with it. Given the small number of clusters in the Ibis Test set, it was analysed as whole with AYB rather than on a tile by tile basis.

**Figure 1:**
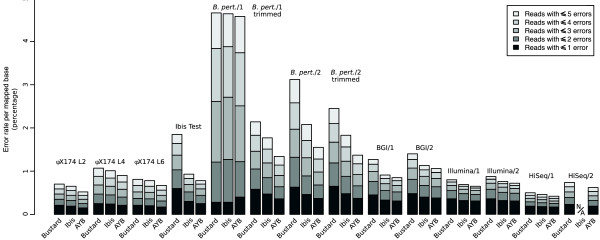
**Comparison of per-mapped-base error rates**. Percentage per-mapped-base error rates for the Bustard, Ibis and AYB base callers compared over several sets of reads of varying read length and chemistry versions. The height of the bars are the percentage of bases that differ from the reference genome, conditioned on reads having been mapped with the stated criteria (a total of 1, . . . , 5 edits relative to the reference). Ibis failed to process the second end of the HiSeq data so no bars are shown.

Both AYB and Ibis improve the number of mapped reads over Bustard by a small amount, with Ibis generally producing a few tenths of a percent more (table 2). The differences between AYB and Ibis are statistically significant only for the *ϕ*X174 L4 and Ibis Test data sets. In contrast, AYB always produces several percent more perfect reads than Ibis, which itself produces several percent more than Bustard. All these differences represent an appreciable fraction of all reads which may have consequences for down-stream analysis since the per-mapped-base error rate for AYB is between 80% and 90% of that for Ibis (82%, 90%, 86% and 85% for the *ϕ*X174 L2, *ϕ*X174 L4, *ϕ*X174 L6 and Ibis Tests sets respectively). The improvement in error rate of AYB over Ibis and Bustard for a variety of mapping criteria is shown graphically in Figure 1, AYB almost always having a lower percentage error than the other base callers.

### Bordetella pertussis, *76 cycle paired-end*

A second comparison was based on a data set comprising an entire lane (100 tiles) of 76 cycle paired-end reads from the coccobacillus *Bordetella pertussis *(data sets denoted *B. pert*./1 and *B. pert*./2 for the two paired ends, respectively), using the complete genome of the Tohoma I strain as a reference. The tiles from this run showed a large variation in the number of clusters, ranging from 345 to 82,000, with the tiles close to the ends of the flow cell containing fewer clusters. The sequence produced was generally of low quality, with Bustard producing an error rate of 50% for the final five bases of the first end of the read-pairs, which suggests that a problem occurred during the sequencing. Oddities in the cross-talk matrix, the channels corresponding to A and C nucleotides being noticeably brighter than those corresponding to G and T, suggest that there may have been illumination problems with one of the lasers. Because of the problems with this run and the presence of polymorphisms relative to the reference genome, it provides a useful comparison between the base callers when problems occur.

Mapping back to the reference revealed a marked difference in base-caller performance (table 2): AYB produced more than four times as many perfect reads as Bustard (1,133k vs. 265k reads) and 1.5 times as many as Ibis (752k reads) for the second end of the read-pairs. AYB produces 56% more mapped reads than Bustard and 13% more than Ibis. The increased number of perfect and close to perfect reads produced by AYB has real consequences for down-stream analysis, with the genome being covered to an average depth of 29.3 × for AYB, 9.5 × for Bustard and 21.7 × for Ibis. Greater coverage means more confident SNP and variant detection, which in turn leads to improved mapping of reads. For the first end of the read, AYB produces a greater number of perfect reads (176k reads) than Bustard (108k) or Ibis (133k) and 59% and 17% more mapped reads than Bustard or Ibis, respectively.

As well as mapping to a reference genome, the length of contigs produced by *de novo *assembly is a useful guide to the quality of reads produced and of relevance in cases where a reference is not available. Applying Velvet [18] to the second end of the *B. pertussis *paired-end reads (kmer length 31; automatic coverage cut off; default options otherwise) produces a N50 contig length of 6690 bases for the AYB reads; the reads from both Bustard and Ibis produce much shorter contigs on average, with N50 lengths of 2029 and 4473 bases, respectively.

**Figure 2:**
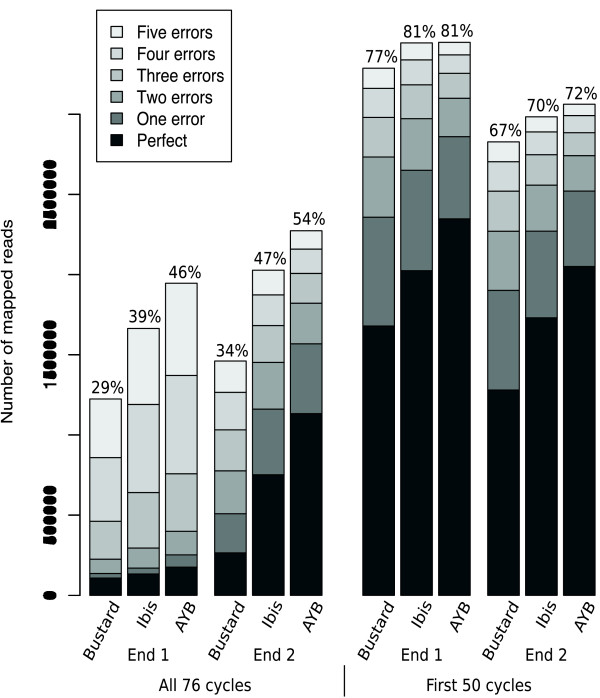
**Number of reads mapped to B**. pertussis. Number of mapped reads and base substitution errors in 4.2 million reads of 76 cycle paired-end *B. pertussis *data relative to a reference genome. The base callers Bustard, Ibis and AYB are compared on ends 1 and 2 of the reads by the number of differences to the reference, to a maximum of five differences, with the total percentage of mapped reads displayed at the top of each bar. Results for both the full reads (left, 'All 76 cycles') and reads trimmed to the first 50 cycles (right, 'First 50 cycles') are shown.

These results are further illustrated in Figure 2, along with the same data trimmed to the first 50 bases to show that AYB still produces more accurate reads even after the later (worst) cycles have been discarded (again, results in table 2). The per-mapped-base error rates are shown in Figure 1, AYB having a lower error rate than Ibis and Ibis having a lower error rate than Bustard for *B. pert*./2 and both trimmed data sets. *B. pert*./1 shows a different pattern with the three callers having about the same error rate, but this should be interpreted in the light of the huge differences in the number of mapped reads produced by the base callers: for reads mapping with either no errors or exactly one error, AYB produced 50% more reads than Ibis, which produced 25% more than Bustard.

### Human NA19240, 45 cycle and 51 cycle paired-end

Much of the pilot data for the 1000 Genomes project [19] has been archived and is publicly available for reanalysis, allowing for a further comparison between the base-callers and showing that AYB can be usefully applied to improve the analysis of existing data. Two sequencing runs for NA19240 (Yoruban daughter) were reanalysed: ERR000479 (9.6 million 45bp paired-end reads, part of ERA000013 by the Beijing Genomics Institute, referred to as sets BGI/1 and BGI/2) and ERR000610 (14.0 million 51bp paired-end reads, part of ERA000023 by Illumina Inc., referred to as sets Illumina/1 and/2). The accuracy of the base-calling was assessed by mapping to the human reference provided by the 1000 Genomes Consortium, based on GRCh37. This genome has variants relative to the sample sequenced but their presence penalises all callers equally. The raw intensities submitted to the archive have apparently been filtered for quality since an abnormally high proportion of the reads map back to the reference when called with Bustard: 86% for the BGI run and 97% for the Illumina run.

Despite the limited scope for improvement, both AYB and Ibis produce slightly more mappable reads than Bustard (table 2): a 1.97% or 1.92% increase respectively for the BGI data and 0.41% or 0.38% increase for the Illumina data averaged over both ends of each set. Large increases over Bustard are observed for the proportion of reads that match the reference exactly, AYB showing a clear lead over Ibis on both sets of data with 14.65% and 5.73% increases over Bustard for the BGI and Illumina sets respectively, compared to increases of 12.74% and 4.56% for Ibis. The superiority of AYB here is surprising as this is not a situation where it would be expected to do particularly better: the number of cycles, and so phasing, is comfortably small in both cases. The read length, vintage and error-rate of the BGI run is consistent with the older "sticky-T" chemistry (incomplete cleavage of the 'T' FLN, leading to an increased concentration in later cycles) and the improvement seen is typical for similar data.

The per-mapped-base error rate for reads produced from all BGI and Illumina data sets by AYB is lower than that of either Bustard or Ibis for a variety of mapping criteria (Figure 1).

### Variant calling of NA19240

Following the guidance for best practice variant calling [20] using the Genome Analysis Tool Kit [21], we implemented a SNP calling pipeline including the full recalibration and realignment of reads. For each base caller, the both BGI and Illumina sets of paired-end reads were combined and then variants for the NA19240 data set were called against the human reference GRCh37. After variant recalibration, 399091 of AYB's 438486 SNP predictions passed the truth sensitivity filter (set to 99.0), compared to 371959 of 407110 for Bustard and 386056 of 413198 for Ibis. Base calls produced by AYB show a 7.3% increase in filtered variant predictions over Bustard, compared with a 3.8% increase shown for variant calls produced by Ibis. The median quality of the calls was 29.9, 29.0 and 27.0 for AYB, Bustard and Ibis, respectively, so calling bases using the AYB base caller results in more variant calls with a higher average recalibrated quality than either of the other two callers.

A set of variant calls for NA19240 produced by deep sequencing with the Complete Genomics technology has been previously published [22] and these allow further verification of the calls produced by each base caller. To permit comparison, all SNP predictions were further filtered to remove those that mapped to genome regions not included in the CG-HQ results, resulting in the removal of 2.3%, 2.2% and 2.3% of predicted SNPs for AYB, Bustard and Ibis, respectively. Validating the remaining predicted variants against the Complete Genomics 'high-quality' set (CG-HQ) [23], the calls produced by AYB show a 6.6% increase over Bustard in validated predictions compared to a 0.4% increase for Ibis. The false discovery rate for AYB was lower than that for either Bustard or Ibis. Full results are shown in table 4.

**Table 4:** Comparison between SNP predictions made by the three base callers and the 'CG-HQ' high-quality set of variant calls from Complete Genomics.

	Match	Δ%	Mismatch	Extra	Complex	Total	FDR
AYB	339908	+6.65	299	36524	13251	389982	8.79 × 10^-4^
Bustard	318725		284	32165	12476	363650	8.90 × 10^-4^
Ibis	319907	+0.37	342	42865	14210	377324	10.68 × 10^-4^

SNP predictions for sites that are SNPs in CG-HQ either 'match' or 'mismatch' depending on whether they agree; for AYB and Ibis matches we also record the percent improvement over Bustard (Δ%). 'Extra' SNP predictions are those for sites that are not present in the CG-HQ data. Predicted SNPs at sites for which the CG-HQ results indicate complex variations from GRCh37, mainly insertions and deletions, are counted as 'complex'. The false discovery rate (FDR) is calculated across matches and mismatches only, since the high level of congruence across base callers in the 'extra' category suggests many of these predicted SNPs may be correct (see text for details). Only variant calls for the autosomal, × or mitochondrial chromosomes are considered.

All three callers predicted a number of SNPs that were not contained in the CG-HQ set: 9.4% of the total for AYB, 8.8% for Bustard and 11.4% for Ibis table 4 (column labelled 'Extra'). Although potentially false positives, these predictions show a good degree of congruence with 40% of the total being common to all three base-callers and 57% being made by at least two base-callers, and so cannot all be discounted as errors. Finally, the median quality of these 'Extra' SNP predictions was lower than those that the CG-HQ set validates ('Match'): 24.1, 24.1 and 20.2 versus 30.0, 29.0 and 27.0 for AYB, Bustard and Ibis, respectively. For comparison, the average quality of SNP predictions at sites where the CG-HQ set predicted a different SNP ('Mismatch') was 24.1, 23.0 and 20.4, respectively. Possible explanations for these discrepancies are variation between the sequence libraries or in the cell lines used [24].

### HiSeq, 101 cycle paired-end

Illumina Inc. made available to us a decimated set of data produced on an Illumina HiSeq machine, comprising 7.8 million paired-end reads (8 lanes with a read length of 101 bases) of human sequence with a *ϕ*X174 spike-in (approximately 0.43% of reads). Ibis was trained separately for each of the 8 lanes, although cross validating revealed little difference in performance since all lanes were from the same sequence library. All statistics reported for Ibis are an average over all 8 models and lanes. The reads were mapped against the reference human genome and that of *ϕ*X174 and, again, the error rates reported are all slightly inflated due to the genomes sequenced having variants relative to the references.

Even on the modern HiSeq chemistry AYB and Ibis improve on the base calls produced by Bustard, although the increase in mapped reads is quite small: 0.32% and 1.18% for the first end for Ibis and AYB, respectively. Larger improvements are seen for the number of perfect reads where both base callers improve on Bustard by several percent (3.75% for Ibis, 6.98% for AYB) so AYB improves on Ibis by almost as much as Ibis improves on Bustard. Greater improvements are seen for AYB on the second end of these reads, with an 11.63% increase over Bustard in the number of perfect reads. We were unable to get Ibis to train on the second end of the reads so results are not available.

For the HiSeq data sets, the per-mapped-base error rate for reads produced by AYB is lower than that of either Bustard or Ibis for a variety of mapping criteria (Figure 1).

### Quality scores

Looking only at per-mapped-base error rates does not tell the whole story of base caller accuracy. Some reads are produced from extremely clean intensities whereas others may have been extracted from very noisy data, and base callers assign each base a quality score to indicate their confidence in that call.

Typically, the quality score is a discrete value related to the estimated probability that the call is correct. Given a set of mapped reads, the actual proportion of bases in error can be found for each (estimated) quality value assigned by the base caller; these proportions can be used to calculate empirical quality values to which the estimated values can be compared to assess their accuracy. If the estimated quality values were perfectly calibrated then they would agree, to within sampling error, with the empirical quality values (a linear relationship with unit slope and zero intercept). For model-based methods like AYB, major discrepancy between estimated and empirical qualities is indicative of poor fit of the model to experimental data. Importantly, knowledge of the accuracy of quality scores, often dependent on per-experiment differences, can be used to calibrate them to give better error probability estimates.

**Figure 3:**
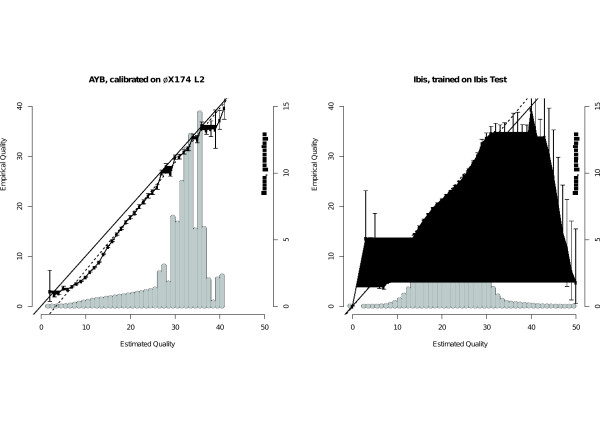
**Quality calibration curves for AYB and Ibis**. Quality calibration curves for AYB (left) and Ibis (right) on the Ibis Test data set of 200K 50 cycle reads. For each base-caller, two graphs are superimposed: a line chart with error bars showing how the empirical quality changes with estimated quality (left axis) and a histogram of how many bases have a given estimated quality (right axis, as a proportion of mapped bases). The error bars represent 99% confidence intervals, obtained by transforming the Wilson interval [32] for the proportion into a quality score. On each graph, straight lines are shown representing perfect correlation between estimated and empirical qualities (solid) and the best linear weighted fit (dashed).

The accuracy of quality scores for AYB, using a calibration function with parameters derived from *ϕ*X174 L2, and Ibis is compared in Figure 3 on the Ibis Test data set. Both base callers produce scores that are fairly reliable for the majority of the quality range, being close to linear with a slight tendency to overestimate confidence in low-quality bases. The scores of extremely high and low quality calls for Ibis are unreliable but, as evidenced by the frequency at which such qualities occur and the width of the 99% confidence intervals, bases with these scores are in the tail of the distribution and so occur rarely. The histogram of the frequency with which a particular quality score is assigned for AYB has a noticeable skew towards higher values whereas the histogram for Ibis has a more Gaussian nature with a lower median.

The Root Mean Squared (RMS) error between the estimated and empirical qualities, weighted by the number of bases assigned to each category, has previously been used to measure quality score accuracy [25]. The RMS criterion is not ideal since it penalises inaccurate qualities for low-quality bases as much as similar errors for the more important high quality bases, and over-estimation of quality is penalised the same as under-estimation despite these two types errors having different consequences for down-stream analysis since over-confidence in a erroneous result will tend to lead to a false positive whereas under-confidence in a true result is conservative. The alternative '*S_p_*' criterion, inspired by information theoretic considerations, has been suggested to overcome these flaws (Richard Durbin, personal communication) and may be thought of as measuring the informativeness of a set of calls with a penalties for being over- or under-confident. Here we define a modified form, ${\bar{S}}_{p}$, constructed so that its maximum occurs when the calibration is exactly correct and its value at this maximum is equal to the average quality score. The criterion is

(1) $${\bar{S}}_{p}=\frac{\sum_{a}n_{a}q_{a}+\frac{10}{\ln10}n_{a}\left(1-10^{\left(q_{a}-q_{a}^{*}\right)/10}\right)}{\sum_{a}n_{a}}$$

where the discrete range of quality values is indexed by *a *and a base caller assigns *n_a _*bases to quality *q_a_*, for which the empirical quality is $q_{a}^{*}$. Notice that the second term of the numerator is negative when the quality is over-estimated and its magnitude increases exponentially with increasing error, whereas the 'bonus' for making a conservative prediction is bounded.

The quality scores produced by the three base-callers for all our sets of data are compared in table 5 using both the RMS and ${\bar{S}}_{p}$ criteria. Qualities were not available for Bustard on the Ibis Test data set and Ibis failed to process the second end of the HiSeq data set. Only Ibis performs uniformly well under both of these criteria, as is expected given that it was trained (calibrated) on the data it was calling or similar (see individual discussion of each data set, above, for details) and should really be compared to recalibrated Bustard and AYB data. The results for AYB may be considered adequate, especially on the four sets (*ϕ*X174 L2, *ϕ*X174 L4, *ϕ*X174 L6 and Ibis Test) which are similar to the one from which its calibration table was obtained (*ϕ*X174 L2), but there is scope for improvement. Bustard's poor scores highlight the desirability of incorporating run-specific recalibration into analysis pipelines; the often poor calibration of its raw scores has been noted previously [21]. The column AYB* in table 5 shows how AYB improves if run-specific calibration is used, the constants for the calibration function in each case being derived from the same subsets of tiles that Ibis was trained on and so producing comparable RMS and ${\bar{S}}_{p}$ scores. Even with our simplistic attempt at run-specific recalibration, see Methods, the quality scores from AYB are consistently more informative than qualities produced by Ibis and this lead can only increase if more sophisticated and accurate recalibration methods were to be applied.

**Table 5:** Assessment of quality score accuracy across all data sets for the three base callers under a variety of criteria; see text for details.

		${\bar{S}}_{p}$			${\bar{S}}_{p}$Max				RMS		*Q*_tot_, *×*10^9^		
	Bustard	Ibis	AYB	AYB*	Bustard	Ibis	AYB	Bustard	Ibis	AYB	Bustard	Ibis	AYB
*ϕ*X174 L2	-35.0	23.4	31.5		26.1	24.7	32.1	12.4	3.6	2.3	1.0	1.0	1.2
*ϕ*X174 L4	9.5	22.3	30.1		25.8	23.0	31.0	8.8	2.4	2.5	1.6	1.5	1.9
*ϕ*X174 L6	-5.6	23.2	31.5		26.8	24.0	32.2	10.1	2.9	2.3	1.3	1.2	1.6
Ibis Test	N/A	23.6	30.7		N/A	24.0	32.0	N/A	1.7	3.2	N/A	0.2	0.3
*B. pert*./1	1.3	21.3	12.0	22.1	20.3	22.5	25.5	8.1	1.8	5.9	1.9	2.8	3.6
*... *trimmed	-1.5	17.0	23.0	24.5	23.2	20.3	27.0	9.1	3.1	4.4	3.8	3.5	4.5
*B. pert*./2	4.9	16.4	23.9	24.2	22.4	20.3	26.6	9.6	1.4	3.7	2.5	3.1	4.4
*... *trimmed	-3.7	14.0	23.6	24.5	22.3	19.9	26.7	9.3	2.6	4.0	3.1	3.0	3.9
BGI/1	-29.0	21.6	0.0	22.9	22.7	22.2	24.0	11.3	2.5	8.7	8.6	8.5	8.8
BGI/2	-9.8	20.6	1.0	22.3	22.3	21.2	23.6	11.1	2.5	8.5	8.2	7.9	8.4
Illumina/1	-1.4	22.8	1.3	23.8	24.0	23.2	24.9	9.8	1.9	8.8	16.7	14.8	16.7
Illumina/2	3.4	22.4	3.1	23.7	23.9	22.8	24.9	9.7	1.9	8.6	16.4	15.7	16.5
HiSeq/1	-0.3	24.9	11.6	25.6	25.9	25.8	26.6	9.6	2.7	7.5	17.4	17.3	17.7
HiSeq/2	1.4	N/A	12.4	24.9	25.5	N/A	26.5	9.2	N/A	7.3	16.1	N/A	16.7

Note that higher values of ${\bar{S}}_{p}$ and ${\bar{S}}_{p}$ Max and lower values of RMS are better. The scores for AYB were calculated using a calibration function with parameters derived from the *ϕ*X174 L2 data set; the scores reported under AYB* are those achieved using run-specific calibration on the larger data sets (see text for details). Quality scores were not available for Bustard on the Ibis Test data set and Ibis failed to train on the second end of the HiSeq data.

The maximum value of ${\bar{S}}_{p}$ assuming perfect calibration, ${\bar{S}}_{p}$ Max, is also shown in table 5 as a measure of the maximum amount of information that could be extracted from the base calls assuming further, probably reference-based, calibration. For these scores, Bustard outperforms Ibis, with the sole exception of the first end of the *B. pertussis *data set, in complete contrast to their relative performance for the other criteria. While Ibis makes fewer base calling errors than Bustard, it does not do such a good job of separating the bases according to confidence and so the total information content of the calls is lower. AYB outperforms both Bustard and Ibis, particularly on the four sets of data that are similar to that which its calibration table was estimated (*ϕ*X174 L2, *ϕ*X174 L4, *ϕ*X174 L6 and Ibis Test).

The 'Total Quality', *Q*_tot_, is a measure of the total information content of the entire set of called bases and is equal to ${\bar{S}}_{p}$ Max multiplied by the number of mapped bases. As it is a sum rather than an average, the *Q*_tot _criterion allows base callers to compensate in bulk for producing low quality calls. The values of *Q*_tot _are also shown in table 5 and have trends that are broadly similar to those for ${\bar{S}}_{p}$ Max, with Ibis generally having a slightly worse scores than Bustard and AYB performing best for all data sets. A notable difference between the ${\bar{S}}_{p}$ Max and *Q*_tot _results is that Ibis has a much better *Q*_tot _score than Bustard on both ends of the *B. pertussis *data, reflecting the increased proportion of mapped reads (see table 2). Ibis has an important advantage over the other base-callers on the RMS and *S_p _*criteria as it was always trained on representative data, and so effectively recalibrated using a reference each time. This advantage is evidenced by the poor performance of Bustard and the improvement in AYB's performance when run-specific calibration is used. These results should not be taken as a indication of the superiority of the quality scores or calibration of Ibis since whatever data was used to train Ibis could also have been used to come up with a good calibration for either AYB or Bustard. While the calibration of neither Bustard nor AYB is exceptional, their performance on the ${\bar{S}}_{p}$ Max and *Q*_tot _criteria, as well as the ${\bar{S}}_{p}$ scores for AYB on the *ϕ*X174 sets of data and after recalibration for other data sets, suggest that bases from both callers can be recalibrated to produce qualities whose informativeness equals or exceeds those from Ibis.

## Conclusions

A particular focus when developing AYB was to make the algorithms robust to problems that might arise during normal use, so it can be used confidently in cases where other base-callers require manual intervention to get the best results. The *B. pertussis *example was presented as such a case; another is data produced using the TraDIS technique [26] where the first few cycles of every cluster consist of known identical sequence, causing algorithms that estimate cross-talk from a single early cycle to fail.

The statistical model underlying AYB has several weaknesses that could be addressed in future work. The model assumes that the descriptive parameters of the sequence process are constant across a tile but this is only going to be approximately true in practice: differences in illumination (e.g. mode scrambler problems) and the relative intensities of the two lasers will affect the cross-talk and background noise; also the expected amount of phasing might be affected by fluctuations in the chemistry. The phasing matrix represents an average over many clusters and the actual amount of phasing at a particular cluster is subject to stochastic variation. The fewer molecules contained in the cluster, the further from the average it is likely to deviate and this can lead to counter-intuitive consequences as a small cluster that has, by chance, undergone little phasing may fit the average model as poorly as one that has undergone a lot of phasing -- clusters could then be penalised despite giving clear signal.

Sequence-like errors, for example mutations introduced during sample preparation, short fragments ligating together or adapter sequence, are essentially invisible to any base-caller and render it impossible to call the original sequence accurately. Other sources of error may not appear sequence-like: for example, microscopic particles of dust can get entangled in a cluster and produce bright artefacts for one or more cycles. Since very bright peaks deviate from the average brightness of the read, AYB penalises these calls heavily and they rarely contribute to the higher quality base-calls; they also reduce the quality of the surrounding calls due to over-correction for pre- and post-phasing. Ideally over-bright peaks would be removed prior to analysis and treated as missing data, with the actual intensity and base-call imputed from the remaining three intensities and the effect the position has on the neighbouring cycles through the phasing correction. A similar idea could be used to deal with clusters where intensities are missing (i.e. unrecorded, perhaps due to image registration problems) for some cycles, producing low quality calls rather than as at present where they are treated as a cycle with four exactly zero intensities.

A final issue that AYB fails to account for is that of heterogeneous clusters of sequence, a common cause of which is two clusters merging into each other during the amplification step, since there is an implicit assumption that each cluster only contains fragments from one particular sequence. The intensities from such clusters appear to be extremely noisy, far above the stochastic background, and AYB's criteria to assess model fit are misled since the effects of both constituent sequences need to be be removed to get the residual noise. Failure to do so means that the calls from the strongest sequence get penalised for badly fitting the model; in particular, cycles where the two constituent clusters have the same base appear much brighter than expected given the intensities from other positions and are thus penalised despite the fact we should be more confident about these calls. In principle heterogeneous sequence could be explicitly estimated for each cluster, the relative brightness being used to separate contributions, but this will result in a loss of power in the majority of cases where the cluster is homogeneous and may not result in high-quality calls otherwise.

Despite being noticeably better than those produced by Bustard, the uncalibrated quality values for AYB are worse than might be desired. Improvements to these could be the subject of further work. Run-specific recalibration leads to significant improvement, with AYB outperforming Ibis. As with Bustard, and indeed all other base callers, we recommend that the qualities produced by AYB should be recalibrated whenever possible as part of the analysis pipeline. The superiority of AYB on both the *S_p_*Max and *Q*_tot _criteria suggest that such recalibration would be fruitful. Where a good quality reference is not available, recalibration based on reads from a spiked-in known genome is a promising approach that could be taken advantage of. Such spike-in data may also help with convergence and improve the estimate of the interaction matrix since it provides a set of reads whose sequence does not have to be estimated.

The speed at which tiles can be analysed is extremely important given the vast amount of data produced by current and future platforms. AYB is much quicker than many competitive base callers, taking only a few minutes to analyse each tile on ordinary computing hardware and of comparable speed to Ibis, the most accurate alternative base caller (table 3).

Even AYB's speed could be prohibitive if computing resources are limited. There are, however, avenues to increase the speed of AYB with possible trade-offs against accuracy. As noted previously, AYB assumes that the sequencing process is constant within a tile and this assumption could be to strengthened to assuming it is constant across multiple tiles or across lanes, a similar assumption to that which Bustard and other base calling programs implicitly make when they train or estimate parameters on a subset of the data. The parameters describing the sequencing process could be estimated from a subset of data and then held fixed so AYB need only perform a base calling step for the majority of the clusters with a considerable reduction in processing time. The major bottleneck for AYB is processing the raw intensities for each cluster, a step that is repeated every iteration and is quadratic in the number of cycles, and speeding up this calculation would greatly accelerate the algorithm. One potential approach would be to assume that the interaction matrix *A *(see Methods) is sparse, the intensities at one cycle only depending on the sequence at nearby cycles for example.

AYB is more accurate than other methods of base-calling. In comparison with the leading competitor, Ibis [9], it generally gives similar or improved performance in the number of mapped reads, and in our tests it always performed considerably better in the number of perfect (error-free) reads (table 2) and almost always achieves a lower per-mapped-base error rate (Figure 1). As the yield from sequencing machines increases, speed of analysis becomes important and our base-calling method offers a unique combination of speed and accuracy. Such a combination is ideally suited for use with more recent 'personal' platforms, such as Illumina's MiSeq [27], which are aimed at smaller institutes and research groups who will be interested in a diverse range of organisms for which a good reference genome is not likely to be available. In addition to its speed and accuracy, AYB has two other desirable properties. First, it does not require training data so calls can be made where a reference sequence is unknown. Second, it uses robust statistical methods to limit undesirable consequences of gross errors in a few clusters.

The AYB base-calling software is written in C and available under the GPL v. 3 licence from http://www.ebi.ac.uk/goldman-srv/AYB/. A set of utilities for extracting and manipulating CIF format intensity data files, under the same licence as AYB, is available from http://www.ebi.ac.uk/goldman-srv/ciftools/.

## Materials and methods

The two major differences between AYB and other base-callers are its empirical model of the sequencing process, potentially allowing the intensities at a given cycle to depend on the entire sequence rather than just a few neighbouring cycles, and its focus on robust algorithms so that sensible base calls are still made even when problems have occurred during a run. Here we describe the underlying statistical model used by AYB, the method of estimation and the techniques used to make the procedure robust.

The foundation of AYB is a mechanistic model of the sequencing process, relating what is observed at each cycle to the underlying sequence of nucleotides. Clusters are analysed in groups, the natural such group being a tile, with the interaction between cycles assumed to be constant and common to all clusters within each group. Other parameters such as the luminescence and the sequence are specific to each cluster. We first describe a simple model of how the observed intensities might be related to the underlying sequence and then show how AYB generalises it.

Each cluster (indexed by *i*) is considered to contain homogeneous sequence, represented by the *base × position *matrix *S_i _*whose (*b*, *j*) entry is one if the base at the *j^th ^*position of the sequence is base '*b*' or zero otherwise. Each column of *S_i _*therefore contains exactly one non-zero entry. The amount of light emitted by a cluster in a given cycle is proportional to the number of FLNs bound to the cluster, which in turn is proportional to the number of molecules in the cluster; this cluster-specific scaling is represented by the scalar *λ_i_*, referred to as the luminescence since it also incorporates a factor representing the intensity of light incident on the cluster and implicitly models variation in incident radiation across the slide.

Due to phasing, the molecules within a cluster lose synchronicity with each other and the relationship between position and cycle becomes blurred; the procession from one cycle to the next of an average cluster is described by the *position × cycle *phasing matrix *P*. Each column of *P *corresponds to one cycle and describes the distribution of sequence positions where the FLNs bind, so the (*j*, *k*) entry is the relative proportion of FLNs bound to position *j *of the sequence on cycle *k *of the sequencing process. As sequencing progresses, the signal decreases as molecules randomly become inactive and stop contributing (dimming) and this is incorporated into *P *by scaling its columns so each sums to the proportion of molecules in the cluster expected to be still active. An ideal *P *would have ones down its leading diagonal with all other elements being zero; a good *P *will be dominated by its diagonal and each column sum will be close to one. Elements of *P *are non-negative, and its column sums are ≤ 1.

Finally, the emissions from each cluster are observed via the four channels and the cross-talk, the relationship between fluorophore emission and what is observed in each channel, is represented by a *channel × base *(4 × 4) matrix *M*. Column *b *of *M *describes the strength of signal in each of the four channels for a unit emission of the FLN *b*. In principle the cross-talk is determined by the physics of system, and so it is assumed to be the same for all cycles.

Putting together all the components of the sequencing process model described above, the observed intensities *I_i _*(a *channel × cycle *matrix) for cluster *i *is related to the underlying sequence by the relationship

(2) $$I_{i}=\lambda_{i}MS_{i}P+N+\varepsilon_{i}$$

where *N *is systematic background noise for all clusters and *ε_i _*is the residual error for the fit to the intensities, an observation of a random variable with expectation zero. Both *N *and *ε_i _*are *channel × cycle *matrices. Note that the number of channels is equal to the number of bases and that the number of positions is equal to the number of cycles, so both *M *and *P *are square matrices. Equation 2 can be expressed to show that the observed intensities are a linear function of the sequence

$$\text{vec}I_{i}=\lambda_{i}\left(P^{t}⊗M\right)\text{vec}S_{i}+\text{vec}N+\text{vec}\varepsilon_{i}$$

where vec is the operator that forms a vector from a matrix by stacking its columns in order, *P^t ^*is the transpose of *P *and ⊗ is the Kronecker product of two matrices. AYB generalises this model by assuming a general linear relationship between the sequence and the intensities,

(3) $$\text{vec}I_{i}=\lambda_{i}A\text{vec}S_{i}+\text{vec}N+\text{vec}\varepsilon_{i}$$

where *A *is the interaction matrix, a (*channel × cycle*) × (*base × position*) matrix describing the effect that specific bases at each cycle have on the intensities for all cycles. Note that *A *allows for the cross-talk to vary between cycles and for the rate of phasing to depend on previous bases.

The statistical model described by equation 3 could be fitted to the raw intensity data using a variety of criteria (maximum likelihood, Bayesian techniques, etc.) but we chose a least squares criterion using an iterative approach. The major reasons for the use of least squares are that analytic solutions exist for many of the steps of the iteration, making it computationally efficient, and that the simple Iteratively reWeighted Least Squares (IWLS) technique can be used to fit the model in a manner robust to contamination [28]. The IWLS approach is similar to Ordinary Least Squares (OLS), seeking to minimise the sum of squared errors over all the clusters, but the squared error for each cluster is weighted and the algorithm proceeds iteratively with the weights being updated between iterations; each iteration is equivalent to the Weighted Least Squares (WLS) criterion, which has an analytic solution. The weights are defined by a function of how well each cluster fits the model relative to the other clusters, so badly fitting clusters (high residual error) get progressively down-weighted. AYB uses the Cauchy function for weighting but many alternatives have been described and are summarised in the subsection on 'Robust Estimation' in *Numerical Recipes *[29].

Given this statistical formulation, the core of the AYB method can be described by the following seven steps, the solution of which will be described in the following sections:

1. Initialise estimates; set all weights to one.

2. Estimate interaction *A *and systematic noise*N*.

3. Estimate cluster-specific luminescence λ*_i_*.

4. Call bases for each cluster, giving sequence *S_i_*.

5. Update weights for all clusters.

6. Iterate steps 2-5 to refine estimates.

7. Assess quality of calls.

### Initialisation

Initialising to good values greatly helps the speed of the AYB algorithm, reducing the number of iterations needed until a good solution is found. A good starting value may be available from previous analyses using the same machine and protocol but, by default, AYB uses the more crude approach of ignoring phasing and dimming and assuming that the cross-talk is the same at all cycles: if *M *is a cross-talk matrix then the initial estimate of the interaction matrix is *A*_0 _= I_D _⊗ *M *where *I_D _*is the identity matrix of dimension *cycle × position*. An initial cross-talk matrix *M *can be found from the intensities of an early cycle of the run [5], making the implicit assumption that phasing does not contribute a significant amount to these observed intensities, but, since cross-talk is primarily determined by physics and has a similar form on different runs and machines, AYB instead initialises *M *to a fixed good value. Systematic noise is initially assumed to be absent.

Setting *A *to *A*_0 _and solving equation 3 for λ*_i_S_i_*, assuming that the systematic and random noise (*N *and ∈*_i_*) are zero, gives a set of corrected intensities from which bases and luminescence can be estimated. The initial estimate of the base at each position is that which has the greatest intensity, and the luminescence of the cluster is the mean of the intensities of the called bases.

### Estimation of the interaction matrix and noise

Equation 3, relating the sequence to the expected intensities, is linear but standard linear regression techniques produce unstable estimates for the interaction matrix; to see why, notice that any permutation of the columns of *A *and rows of *S_i _*leaves the intensities unchanged and so, when estimating the interaction is iterated with base-calling, the solution can jump between permutations. We use generalized Tikhonov regularisation [30] in a weighted least squares solution for *A *and *N *to favour there being no permutation of *A *and *S_i_*.

Defining the adjusted interaction matrix *A*' and adjusted sequence *S*' by

$$A^{\prime }=\left(A,\text{vec}N\right)S^{\prime }=\left(\begin{array}{c} \lambda_{i}\text{vec}S_{i}\\ 1 \end{array}\right)$$

then the regularised weighted least squares estimate $\hat{A^{\prime }}$ for *A*', and thus for *A *and *N*, is

$${\hat{A^{\prime }}}^{t}={\left(p{\text{I}}_{\text{D}}+\underset{i}{\sum }w_{i}S_{i}^{\prime }{S_{i}^{\prime }}^{t}\right)}^{-1}\left(pB+\underset{i}{\sum }w_{i}S_{i}^{\prime }\text{vec}I_{i}^{t}\right)$$

where I_D _is an identity matrix of the appropriate dimension, *p *is a constant specifying the strength of regularisation, and $B=\left(A_{0}^{t},0\right)$ with **0 **being a vector consisting of zeros. For convenience, the solution is regularised towards the value used to initialise the algorithm, although this is not a requirement and other choices may be more desirable.

There is an arbitrary scaling factor implicit in equation 3, corresponding to the scale that the luminescence is measured on. If all elements of interaction matrix are doubled and every λ*_i _*is halved, then the expected intensities are unchanged. This ambiguity is resolved by scaling the interaction matrix so that its determinant is one.

### Estimation of luminescence

The estimation of luminescence for each cluster can be found simply by least squares, using the same criterion as that used to estimate the interaction and systematic noise. The least squares estimate of the luminescence $\hat{\lambda_{i}}$ of each cluster is

$$\hat{\lambda_{i}}=\frac{\text{vec}S_{i}^{t}A^{t}\text{vec}I_{i}}{\text{vec}S_{i}^{t}A^{t}A\text{vec}S_{i}}$$

which is an ordinary least squares estimate since the weights used for the estimation of the interaction matrix are cluster-specific and so cancel.

### Base calling

As well as being linear in the interaction matrix, the observed intensities in equation 3 are also a linear function of the sequence. As described, equation 3 assumes that each element of the random error is independent and identically distributed (IID) but this is not found to be the case in real data (results not shown). The violation of the IID assumption is not a problem when estimating the interaction matrix, since these estimates are produced from a large number of independent clusters and so the random error is small, but is much more significant when trying to estimate the sequence since there are many fewer, dependent, observations. Forcing the IID assumption onto the random noise produces poor base calls (results not shown) and so correlation between the elements of *ε_i _*must be taken into account and the sequence that minimises the generalised least squared error must be found.

Intensities after correction for interaction and systematic noise are defined to be

$$C_{i}=A^{-1}\text{vec}\left(I_{i}-N\right)$$

and the sequence that minimises the generalised least square error of equation 3 also minimises the generalised least square error of the same relation written in terms of the corrected intensities *C_i_*. This latter formulation is more convenient to work with. Finding the minimum generalised least square is a type of constrained binary quadratic programming problem and so difficult to solve exactly. Instead of solving directly, we make the additional assumption that each read position only depends on its immediate neighbours and so most positions are conditionally independent of each other. This dependence structure requires that the inverse of the covariance matrix is block tridiagonal; that is, it consists of a grid of *base × base *matrices and this grid is tridiagonal. The maximum likelihood estimate of the required covariance matrix is found by numerical optimisation (conjugate gradient algorithm) of the log-likelihood function parametrised in terms of the Cholesky factorisation of the matrix; full details are contained in Additional file 1.

The structure of the inverse covariance matrix means that the log-likelihood for a cluster *i *having the sequence *s*_0_, *s*_1_, . . . , *s_n _*can be written as $k+\sum_{j=1}^{n}a_{j,s_{j},s_{j+1}}$ for suitably chosen tensor *a_f,g,h _*and a constant *k*, and so is a one-dimensional Gibbs field. The classic Viterbi and Forward/Backward dynamic programming algorithms can be used to find the most likely sequence or the posterior distribution of bases at each position.

### Updating weights

The weighting of the clusters plays an important part in making the AYB method robust to contamination and other misleading observations, reducing their influence on the parameter estimates. The weight for each cluster is calculated, after all model parameters have been fitted, using the Cauchy function so *w_i _*= 1*/*(1 + *L_i_/*2*μ*) where *L_i _*is the least square error for cluster *i *and *μ *is a measure of the central trend of the *L_i _*(their mean, for example). In contrast to OLS, where every cluster would receive a weight of one, this weighting function means that only perfect observations, those with a least square error of zero, receive full weight whereas worse-fitting observations receive progressively lower weights.

### Iteration and termination

As the luminescence and individual bases are estimated (steps 3-4 above) using a different criterion to that used to estimate the interaction matrix (step 2), the least squares error is not guaranteed to decrease when the parameter estimation and base calling steps are iterated. Theoretically this could lead to problems with convergence but this was not found to be the case, a small number of cycles sufficing to get good estimates. Numerical experiments suggest that three to five iterations are sufficient, with little change in accuracy for addition iterations (results not shown).

### Assessment of quality

To differentiate between good and bad reads, each base is assigned a quality score -- a measure of the probability that it has been correctly called. Commonly these are reported as Phred scores: *Q*_Phred _= *-*10 log_10 _*e*, where *e *is the probability of a base being incorrect [31]. It is trivial to convert these scores to and from probabilities, so one only needs to assess the probability of each call being incorrect. We treat base calling quality assessment as a model selection problem, choosing between the four models 'A', 'C', 'G' and 'T' for each cycle of each cluster, and apply Bayes theorem to get the posterior probability *p_i_*;*_bj _*that the base at position *j *in cluster *i *is *b*:

(5) $$p_{i;bj}=\frac{\pi_{b}f_{i;bj}}{\sum_{x\in \left\{A,C,G,T\right\}}\pi_{x}f_{i;xj}}$$

where $f_{i;xj}={\left(1+L_{i;xj}\right)}^{-\left(1+n^{*}\right)/2}$ is the (scaled) probability density of the observed intensities, *L_i_*;*_xj _*is the least square error for cluster *i *given that the base at position *j *is *x*, and *π_x _*is the prior probability of base *x*. The required least squared errors can be calculated for all bases and positions simultaneously using a Forwards/Backwards modification of the Viterbi algorithm used to find the best sequence of bases (step 4 above). The particular form of *f_i_*;*_bj _*comes from assuming that the random error has an elliptical distribution defined by the Cauchy function, in keeping with our choice of weighting function for the IWLS estimation. The parameter n* should normally be equal to the dimension of the elliptical distribution but, instead, we use the median of the observed least squared errors as this helps to correct for skews in the distribution.

Reads can also be wrong for reasons other than base calling error, a good example of this being polymerase errors during sample preparation. The net effect of these 'generalised' errors is to bound the maximum possible quality of a call and they are incorporated into AYB's quality scores as a constant probability of error independent of the probability that the base was called incorrectly. The final probability that a base is correct, incorporating the notion of generalised error, is $p_{i;bj}^{*}=\left(1-\varepsilon \right)p_{i;bj}$ where *ε *is the constant probability of a generalised error. The corresponding quality score is $Q_{i;bj}^{*}=-10\log_{10}\left(1-p_{i;bj}^{*}\right)$. Despite the methods incorporated into AYB being robust and attempts being made to compensate for effects of unusual clusters, the quality scores produced may not be accurate because of differences between AYB's assumptions and how the sequencing machines actually operate: not every source of error can be incorporated into the model and the various distributional assumptions made can only be approximate. To improve concordance, quality scores may be calibrated to real data [31] using some form of table look-up or calibration function. There are many good methods to calibrate quality scores [21,25,31] but, for the purposes of the comparisons in this paper, we use a simple linear calibration function for AYB and note that it could be improved upon.

The calibrated quality $Q_{i;bj}^{\text{cal}}$ of the called base *b_j _*at position *j *in cluster *i *is defined by

$$Q_{i;bj}^{\text{cal}}=\alpha_{b_{j-1},b_{j+1}}+\beta Q_{i;bj}^{*}$$

where the *base × base × base *table *α *and the constant *β *are chosen to agree with representative data from a real sequencing run. We expect these parameters to vary with difference machines, chemistries and experimental protocols, and typical values based on the sets of data analysed within this paper are distributed with the AYB software. We provide a tool to produce bespoke constants given a set of mapped data; however, we reiterate our recommendation that quality scores should be recalibrated using a reference whenever possible.

## Competing interests

None declared.

## Authors' contributions

TM designed the study, derived the statistical models and implemented them in software, carried out the comparisons and performed the statistical analysis, and drafted the manuscript. NG conceived and helped to design the study and helped to draft the manuscript. Both authors read and approved the final manuscript.

## Supplementary Material

Additional file 1 **Fitting a block tridiagonal information matrix by ML. Additional data file 1 is a document describing how to find the maximum likelihood estimate of the inverse covariance matrix when several of its components are conditionally independent**. http://genomebiology.com/imedia/5688411755799342/supp1.pdf. Click here for file
