# Supplementary material for: All Your Base: a fast and accurate probabilistic approach to base calling
Source: Genome Biol. 2012 Feb 29;13(2):R13. doi: 10.1186/gb-2012-13-2-r13 (PMC4053729; doi:10.1186/gb-2012-13-2-r13)
Supplement: Additional file 1 — Fitting a block tridiagonal information matrix by ML. Additional data file 1 is a document describing how to find the maximum likelihood estimate of the inverse covariance matrix when several of its components are conditionally independent. http://genomebiology.com/imedia/5688411755799342/supp1.pdf. [file gb-2012-13-2-r13-S1.PDF]

Additional material for Massingham and Goldman.
All Your Base: a fast and accurate probabilistic approach to base calling

Fitting a block tridiagonal information matrix by ML

The aim of this appendix is to show how to find the Maximum Likelihood (ML) estimate of the variance matrix of a multivariate normal distribution with known mean when each component is known to be conditionally independent of the others given the values of a few neighbours. This conditional independence is made obvious in the information matrix (also known as the precision matrix) of the random variable since the relevant entries must be zero. Specifically we will be looking at matrices that are block tridiagonal since the cycle structure of the base calling problem partitions the variance into a grid of 4×4 blocks and each cycle only depends on its neighbours.

In the first section we prove a useful theorem about a class of matrices; in the second section we show how this can be used the find the required maximum likelihood estimate using numerical optimisation techniques.

1 Ragged and shaggy square matrices

Definition A square matrix M is lower-ragged if, for every row i, there is a column m_i such that $M_{ij} = 0 \ \forall j < m_i$ and the sequence $m_1, m_2, \ldots$ is non-decreasing. The form of a lower-ragged matrix is the binary matrix F where $F_{ij} = 0 \ \forall j < m_i$ and one otherwise.

Lemma 1.1 *A submatrix of a lower-ragged matrix is also lower-ragged*

Proof Trivial. Pick out relevant m_i from the sequence and renumber. Order is preserved.

Lemma 1.2 *If R is lower-ragged with form F and U is an upper-triangular matrix, then the matrix product RU is also lower-ragged with form F.*

Proof Since U is upper-triangular, $U_{kj} = 0$ whenever $k > j$. Since R is lower-ragged, $R_{ik} = 0$ whenever $k < m_i$. Hence the (i, j) element of the matrix product $(RU)_{ij} = \sum_k R_{ik} U_{kj}$ must be zero whenever $j < m_i$. Therefore RU is lower-ragged with form F.

Theorem 1.3 *If a lower-ragged matrix has a LU decomposition, then the lower-triangular half is lower-ragged with the same form.*

Tim Massingham, European Bioinformatics Institute

Proof By induction. Trivially true for 1×1 matrix. Assume true for matrices up to size i. Consider a matrix of size $i+1$ and decompose into blocks such that A and D are square:

$$\begin{aligned}\begin{pmatrix} A & B \\ C & D \end{pmatrix} &= \begin{pmatrix} I & 0 \\ CA^{-1} & I \end{pmatrix}\begin{pmatrix} A & 0 \\ 0 & D - CA^{-1}B \end{pmatrix}\begin{pmatrix} I & A^{-1}B \\ 0 & I \end{pmatrix} \\ &= \begin{pmatrix} L & 0 \\ CU^{-1} & L' \end{pmatrix}\begin{pmatrix} U & L^{-1}B \\ 0 & U' \end{pmatrix}\end{aligned}$$

where A has decomposition LU and $D - CA^{-1}B$ has decomposition $L'U'$, L being lower-ragged with the same form as A. If D is lower-ragged then, for any row i with $m_i > 0$, the corresponding rows of C must be entirely zero and so $D - CA^{-1}B$ has the same form as D; hence so does its LU decomposition. The matrix C is lower-ragged. Since the inverse of an upper triangular matrix is also upper-triangular, then CU^{-1} is lower-ragged with the same form as C by lemma 1.2

Similarly, an upper-ragged matrix can be defined such that the transpose of an upper-ragged matrix is lower-ragged. If an upper-ragged matrix has a LU decomposition, then the upper-triangular half of the decomposition is upper-ragged with the same form.

Further, let a shaggy matrix be a matrix that is both upper-ragged and lower-ragged; a shaggy matrix has an LU decomposition where both the upper- and lower-triangular halves retain the same form.

Corollary 1.4 *A symmetric positive-definite block tridiagonal matrix has a block tridiagonal Cholesky decomposition.*

Proof A block tridiagonal matrix is a shaggy matrix. The previous lemma applies, noting that $D - CA^{-1}C^t$ is the Schur complement of A and must be positive definite since the original matrix is.

2 Finding the maximum likelihood estimate of Ω

The log-likelihood $L(\Omega)$ of observations x_i for $i \in \{1, \ldots, n\}$ with information matrix Ω is

$$\begin{aligned} L(\Omega) &= K - \frac{1}{2}\sum_i x_i^t \Omega x_i + \frac{n}{2}\log\det\Omega \\ &= K - \frac{1}{2}\operatorname{tr} x_i x_i^t \Omega + \frac{n}{2}\log\det\Omega \\ &= K - \frac{n}{2}\operatorname{tr} V\Omega + \frac{n}{2}\log\det\Omega \qquad (1) \end{aligned}$$

where $V = \sum_i x_i x_i^t$. We wish to maximise the likelihood such that Ω is a positive definite matrix with a block tridiagonal structure — blocks only depend on other blocks through their neighbours. Since Ω must be symmetric positive definite and maximum likelihood estimators are transformation invariant, it is convenient to rewrite equation 1 in terms of its Cholesky decomposition: $\Omega = U^t U$

$$\begin{aligned} L(U) &= K - \frac{n}{2} \operatorname{tr} V U^t U + \frac{n}{2} \log \det U^t U \\ &= K - \frac{n}{2} \operatorname{tr} V U^t U + n \log \det U \end{aligned} \tag{2}$$

which ensures that any estimate of Ω will be symmetric positive definite provided the estimated U is a valid Cholesky factor (its diagonal is strictly positive). For those elements where the derivative is non-zero, i.e. where being shaggy does not require the element of U and hence the derivative with respect to that element to be zero, the derivative of the log-likelihood is

$$\begin{aligned} \left(\frac{dL(U)}{dU}\right)^t &= nF \circ \left(U^{-t} - UV\right) \\ &= n \left(\operatorname{diag} U^{-t} - F \circ UV\right) \end{aligned} \tag{3}$$

where F is the form of U (upper triangular and upper shaggy). Note that, by convention, the (i, j) element of $dL(U)/dU$ is $dL(U)/dU_{ji}$ (the transpose of the order in U). It is unnecessary to explicitly invert U since the diagonal elements of its inverse are the inverses of its diagonal elements.

2.1 Solving the estimating equation

The maximum likelihood estimate of U can be found numerically by multidimensional optimisation of equation 2. The Polak-Ribière conjugate gradient algorithm was chosen since first derivatives of the log-likelihood function are available (equation 3) but the dimension of the optimisation is large and, while second derivatives can be calculated in theory, simple Newton-style methods are impractical due to memory and performance constraints. The gradient of the log-likelihood, and hence the search direction in the conjugate gradient algorithm, satisfy the upper triangular and block tridiagonal structure so, starting at a feasible initial point U_0, all subsequent refinements to the solution also have this structure.

In each iteration of the conjugate gradient algorithm, a linear sub-problem must be solved: finding the value of λ which maximises $L(U_i + \lambda D_i)$ where U_i is starting point for iteration i and D_i is the search direction. Let the maximal value occur at $\widehat{\lambda}$. As noted, the matrix $S_i(\lambda) = U_i + \lambda D_i$ is both upper triangular

and block tridiagonal for all values of λ but care must be taken when solving the linear sub-problem to ensure that $S_i(\widehat{\lambda})$ remains a valid Cholesky factor. It is sufficient to ensure that the diagonal elements of $S_i(\widehat{\lambda})$ are strictly positive and this can be achieved by constraining the range of λ to the interval $[0, c)$ where $c = \min_{i\,:\,D_{ii}<0} -U_{ii}/D_{ii}$. Since the determinant of a triangular matrix is the product of its diagonal elements, the log-likelihood must tend to $-\infty$ if any diagonal element of U tends to zero. Applying the intermediate value theorem followed by Rolle's theorem is sufficient to show that there must be a maximum within the interval for λ. The transformation $\lambda' = -\log(1 - \lambda/c)$ is useful to ensure that line-search respects the boundaries of the interval by removing the asymptote c to $\lambda' = \infty$ and so preventing undesirable behaviour that would otherwise occur in its neighbourhood.

Substituting into equation 2 and making the necessary definitions, the log-likelihood for the linear sub-problem can be rewritten

$$\begin{aligned} L(U_i + \lambda D_i) &= K - \frac{n}{2}\operatorname{tr} V(U_i + \lambda D_i)^t(U_i + \lambda D_i) + n \log\det(U_i + \lambda D_i) \\ &= K'(U_i) + a\lambda + b\lambda^2 + \sum_i \log\left(1 + \lambda r_i\right) \end{aligned}$$

which is substantially cheaper to evaluate for specific values of λ when U_i is constant.
